# Roux-en-Y Gastric Bypass Surgery-Induced Weight Loss and Metabolic Improvements Are Similar in TGR5-Deficient and Wildtype Mice

**DOI:** 10.1007/s11695-018-3297-6

**Published:** 2018-05-16

**Authors:** Zheng Hao, R. Leigh Townsend, Michael B. Mumphrey, Thomas W. Gettys, Sangho Yu, Heike Münzberg, Christopher D. Morrison, Hans-Rudolf Berthoud

**Affiliations:** 1 Neurobiology of Nutrition & Metabolism Department, Pennington Biomedical Research Center, Louisiana State University System, 6400 Perkins Road, Baton Rouge, LA 70808 USA; 2 Nutrient Sensing and Adipocyte Signaling Laboratory, Pennington Biomedical Research Center, Louisiana State University System, Baton Rouge, LA USA

**Keywords:** Obesity, Diabetes, Body weight, Energy expenditure, Hepatosteatosis

## Abstract

**Background and Purpose:**

Roux-en-Y gastric bypass surgery (RYGB) remains one of the most effective treatments for obesity and type 2 diabetes. Despite this, the mechanisms through which it acts are still not well understood. Bile acid signaling through the transmembrane G-protein-coupled receptor TGR5 has been shown to have significant effects on metabolism and has recently been reported to be necessary for the full effects of vertical sleeve gastrectomy (VSG), a bariatric surgery with similar effects to RYGB. The goal of the current study is therefore to investigate the role of bile acid signaling through TGR5 to see if it is necessary to obtain the full effects of RYGB.

**Methods:**

High-fat diet-induced obese TGR5^−/−^ and wildtype mice (WT) were subjected to RYGB, sham surgery, or weight matching (WM) to RYGB mice via caloric restriction. Body weight, body composition, food intake, energy expenditure, glucose tolerance, insulin sensitivity, and liver weight were measured.

**Results:**

Although the difference in fat mass 20 weeks after surgery between RYGB and sham-operated mice was slightly reduced in TGR5^−/−^ mice when compared to wildtype mice, loss of body weight and fat mass from preoperative levels, reduction of food intake, increase of energy expenditure, and improvement in glycemic control were similar in the two genotypes. Furthermore, improvements in glycemic control were similar in non-surgical mice weight-matched to RYGB.

**Conclusions:**

We conclude that bile acid signaling through TGR5 is not required for the beneficial effects of RYGB in the mouse and that RYGB and VSG may achieve their similar beneficial effects through different mechanisms.

**Electronic supplementary material:**

The online version of this article (10.1007/s11695-018-3297-6) contains supplementary material, which is available to authorized users.

## Introduction

Bariatric surgery continues to be the most effective tool to treat severe obesity, but the mechanisms underlying its beneficial effects are not well understood. Although a number of specific candidate mechanisms have been proposed, there is no conclusive evidence for their importance. The increased secretion and circulating levels of the lower gut hormones GLP-1 and PYY (3-36) that are routinely observed after both Roux-en-Y gastric bypass (RYGB) and vertical sleeve gastrectomy (VSG) in both humans and rodents continue to be a major candidate [1–5], but direct long-term interference with GLP-1 signaling was unable to change the ability to reduce body weight and improve glycemic control of both surgeries in mice [4, 6–8].

Recent insights into bariatric surgery-induced changes in bile acid metabolism have suggested that changes in bile acid signaling may be crucial for the beneficial effects of VSG not only in weight loss [9, 10] but also in improvements of glycemic control in mice [11]. Bile acid signaling through both the nuclear receptor FXR and the membrane receptor GPBAR-1/TGR5 expressed in a number of organs and tissues is thought to exert physiologically important effects on the gut, white (WAT) and brown (BAT) adipose tissue, liver, muscle, pancreas, heart, and brain [12–15]. Specifically, bile acid signaling through TGR5 can increase the secretion of GLP-1 from L-cells in the gut [16], energy expenditure through increased BAT thermogenesis and WAT browning [17], and insulin secretion through direct actions on the β-cell [13] and prevent inflammation and hepatosteatosis [18, 19]. Increased serum bile acid concentrations have been shown both after RYGB and VSG in humans [20–26] and rodent models [9, 11, 27]. Furthermore, bile diversion to the lower small intestine in obese mice increases certain circulating bile acids and leads to the same powerful body weight loss as RYGB [28]. Therefore, we hypothesized that bile acid signaling through the TGR5 receptor is not only required for the full beneficial effects of VSG as recently reported in murine studies [9, 11], but also for the full beneficial effects of RYGB. Although VSG is rapidly becoming the most often performed bariatric procedure, the two procedures are very different and likely engage at least some different sets of mechanisms [29]. In addition, in obese humans, long-term success has been better documented for RYGB than with VSG, and some studies find RYGB to be more effective. In murine models, RYGB is far more efficient in producing sustained reduction of body weight below preoperative levels [30], and the outcome regarding weight reduction of the two VSG studies using TGR5-deficient mice was different. While one study found no difference between TGR5-KO and wildtype mice in the weight loss response to VSG [11], the other study reported no weight loss relative to sham surgery controls in TGR5-KO mice at 14 weeks after VSG surgery [9].

Therefore, to directly test the hypothesis that TGR5-signaling is required for the full beneficial effects of RYGB, we performed RYGB in high-fat diet-induced TGR5-KO and WT mice.

## Materials and Methods

### Experimental Overview

A cohort of 26 TGR5-KO and 30 WT mice were exposed to a two-choice high-fat and regular chow diet for 9 (KO) and 15 (WT) weeks and mice of each genotype were stratified into three groups, Roux-en-Y gastric bypass surgery (RYGB, *n* = 10/11), sham surgery (sham, *n* = 10/11), and weight-matched to RYGB by calorie restriction (WM, *n* = 6/8). Body weight was measured before and after surgery every 2–3 days for a total of 20 weeks and then the animals were euthanized and tissues harvested. Body composition was measured with NMR before and every 2 weeks after surgery. Food intake was measured before surgery and on days 1–10 and 60–74 after surgery. Energy expenditure was measured at 2–3 weeks and 16–17 weeks after surgery. Glucose tolerance was measured at 4 weeks and insulin tolerance at 6 weeks after surgery. No other invasive tests such as large volume blood sampling were performed to avoid unintended weight loss.

### Animals and Diets

Male C57BL/6J Gpbar1^−/−^ mice (referred to here as TGR5-KO mice) were originally obtained from Merck Research Laboratories (Kenilworth, NJ) and generated for the present study from a breeding colony. A separate cohort of male C57BL/6J mice purchased from Jackson Laboratories served as wildtype (WT) controls. Starting at about 6 weeks of age, all mice were exposed to a two-choice diet consisting of high-fat (kcal%: Carb, 20; Fat, 60; Prot, 20, Diet D12492, Research Diets, New Brunswick, NJ) and low-fat regular laboratory chow (kcal%: Carb, 58; Fat, 13; Prot, 28.5, Diet 5001, Purina LabDiet, Richmond, IN) for the duration of the experiment, except for periods in the metabolic chamber when they were exposed to only high-fat diet. The rationale for the two-choice diet was twofold; firstly, it better mimics the human situation, and secondly, we found that mice eat relatively more chow immediately after RYGB to maintain motility of the small gastric pouch. Mice were kept individually on corncob bedding except for the periods of food intake measurements, when they were on grid floors. All animals were kept in climate-controlled rooms at a slightly elevated room temperature of 23–24 °C and a 12/12 h light-dark cycle (lights on at 7 am), except for periods near thermoneutrality (29 °C) in metabolic chambers. All mice used for the study were genotyped at the end of the experiment to verify their WT or Gpbar1^−/−^ identity (Supplementary Fig. 1).

Animal care and experimentation was approved by the Institutional Animal Care and Use Committee and strictly followed rules and guidelines provided by the American Physiological Society and NIH.

### RYGB, Sham Surgery, and Weight Matching

RYGB was carried out according to a protocol described previously [31]. Briefly, in a jejuno-gastric anastomosis, the cut end of the mid jejunum was connected with a very small gastric pouch and the other end of the cut jejunum was anastomosed to the lower jejunum, resulting in a 5–6 cm long Roux limb, a 9–11 cm long biliopancreatic limb, and a 20–25 cm long common limb. Sham surgery consisted of laparotomy only, without transection of jejunum and stomach. Mice weight-matched to the RYGB group were restricted to about 50–70% of the caloric intake of the RYGB group. Pre-weighed amounts of food (kcal: ~ 93% high-fat, ~ 7% chow) were given once per day during the light period.

### Measurement of Body Weight, Body Composition, and Food Intake

Body weight was measured every 2–3 days for RYGB and sham mice and every day for WM mice. Body composition was measured before and every 2 weeks (± 4 days) after surgery with a Minispec LF 90 NMR Analyzer (Bruker Corporation, The Woodlands, TX). Adiposity index was defined as fat mass divided by lean mass.

Food intake was measured for 4–7 days before and 4–10 days after surgery, as well as for 10 consecutive days at 9 weeks after surgery. Total food intake in kcal was derived from intake of high-fat diet (5.24 kcal/g) and regular chow diet (3.02 kcal/g) and by taking spillage into account. Chow preference was calculated as the percentage of total food intake in kcals obtained from regular chow diet.

### Measurement of Energy Expenditure, RER, and Locomotor Activity

Energy expenditure, RER, and locomotor activity were measured at the end of the weight loss period (12–25 days after surgery, referred to as 3 weeks) and during the stable weight phase (111–125 days after surgery, referred to as 17 weeks) in metabolic chambers (Phenomaster/Labmaster, TSE Systems, Germany). All mice were first adapted to eating food from hanging baskets in training cages for 4–6 days. Mice that had difficulty eating from hanging baskets were floor-fed. Energy expenditure was measured at two ambient temperatures, normal room temperature at 23 °C for 3 days and near thermoneutrality at 29 °C for 3 days. Mice were adapted for at least 1 day to each condition before taking measurements. Energy expenditure is reported both unadjusted in kcal/mouse and adjusted for lean and total body mass. Locomotor activity was measured in numbers of beam breaks in the X and Y planes (7 mm spatial resolution, 10 ms temporal resolution).

### Glucose and Insulin Tolerance Tests, Fasting Insulin and Leptin

Glucose tolerance was assessed at 30 ± 4 days (referred to as 4 weeks) after surgery by injecting α-D-glucose (1.5 mg/kg, 30% *w*/*v* in sterile saline, i.p.) and measuring blood glucose from the tail vein before and at 15, 30, 60, and 120 min after injection, with glucose strips and a glucometer (Onetouch Ultra Strips and Onetouch Ultra Glucometer, LifeScan INC, Milpitas, CA). Glucose tolerance tests were conducted between 09:00 and noon, after 3–5 h of food deprivation.

Insulin tolerance was assessed at 43 ± 4 days (referred to as 6 weeks) after surgery by injecting insulin (0.6 U/kg in sterile saline, i.p., Novolin R, Novo Nordisk, Bagsvaerd, Denmark) and measuring blood glucose as above.

At 143 ± 4 days after surgery, mice were food deprived for 3–5 h and euthanized by decapitation. A few drops of trunk blood were collected and blood glucose was immediately tested using glucose strips as above. An additional 500 μl of trunk blood was collected, treated with 83.5 μl EDTA (Sigma, St. Louis, MO) and a protease inhibitor cocktail (1.5 μl of each of the following: protease inhibitor, Sigma, St. Louis, MO; DPP-IV inhibitor, EMD Millipore, St. Charles, MO; Prefabloc SC, Roche, Indianapolis, IN), and immediately centrifuged at 4 °C and 3000 RPM for 10 min to separate the plasma from the whole blood. Plasma aliquots were frozen in liquid nitrogen and stored at − 80 °C prior to processing. Plasma was subjected to ELISA for measurement of insulin concentrations (MADKMAG-71K Milliplex map mouse adipokine magnetic bead panel—endocrine multiplex assay, EMD Millipore, St. Charles, MO).

### Statistical Analysis

Differential changes in weight, fat mass, and adiposity index between RYGB and sham were analyzed with Student’s *t* tests and considered significant at *p* < 0.05. Food intake, energy expenditure, locomotor activity, RER, insulin and glucose tolerance AUC, and fasting plasma assays were analyzed with two-way ANOVA using treatment group and genotype as between-subject variables. Benjamini-Hochberg corrected pairwise *t* tests with false discovery rate set at 0.05 were used for specific group comparisons. All data are reported as mean ± SEM.

## Results

### RYGB Has Similar Effects on Body Weight, Body Composition, Food Intake, and Energy Expenditure in WT and TGR5-KO Mice

**Fig. 1:**
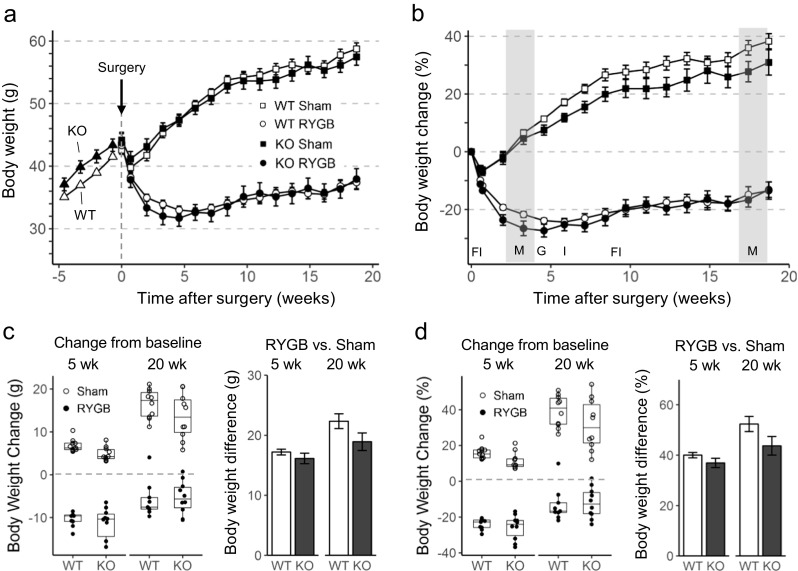
Effect of RYGB or sham surgery on body weight in TGR5^−/−^ (KO) and wildtype (WT) mice. **a**, **b** Effect on absolute body weight and percent body weight change over the 20-week observation period. Effect of high-fat diet on body weight of KO and WT mice for 5 weeks before surgery is also shown in **a**. Timing of measurements of food intake (FI), metabolism (M, shaded areas denote time spent in TSE chambers), glucose tolerance (G), and insulin tolerance (I) is shown in **b**. Means ± SEM, *n* = 9–11. **c** Change of body weight from pre-surgical levels and difference between RYGB and sham surgery at 5 and 20 weeks after surgery. **d** Percent change of body weight from preoperative levels and difference between RYGB and sham surgery at 5 and 20 weeks after surgery

**Fig. 2:**
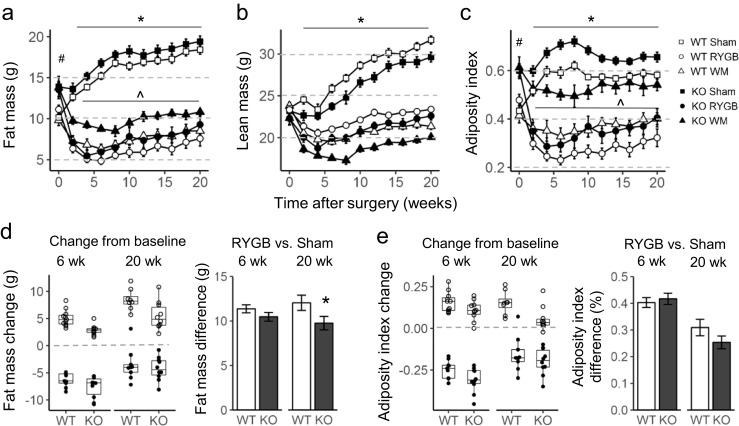
Effect of RYGB, sham surgery, or caloric restriction (to match weight after RYGB) on body composition in TGR5^−/−^ (KO) and wildtype (WT) mice. **a**–**c** Effect on fat mass, lean mass, and adiposity index (fat mass/lean mass). Means ± SEM, *n* = 6–11 mice. *p < 0.01, RYGB vs. sham both genotypes; ^#^*p* < 0.001, KO vs. WT; ^*p* < 0.001, based on repeated measures ANOVA, RYGB vs. WM, both genotypes. **d**, **e** Change of fat mass (D) and adiposity index (E) from baseline and differences between RYGB and sham surgery at 6 and 20 weeks after surgery. *p < 0.05, WT vs. KO

Exposure to the two-choice high-fat diet before surgery led to steady gain of body weight and fat mass in both genotypes but because TGR5^−/−^ are more prone to diet-induced obesity, they reached preoperative body weight after only 9 weeks, while it took WT mice 15 weeks on the high-fat diet. At the time of surgery, body weight was slightly but not significantly higher in TGR5^−/−^ compared with WT mice (44.0 ± 0.9 vs. 42.6 ± 0.4 g, n.s.) (Fig. 1a). However, fat mass gain was greater in TGR5-KO mice so that at the time of surgery, fat mass and adiposity index were slightly but significantly higher compared with WT mice (fat mass 13.7 ± 0.5 vs. 10.4 ± 0.3 g, *p* < 0.001; adiposity index 0.60 ± 0.02 vs. 0.44 ± 0.02; *p* < 0.001) (Fig. 2a, c).

All mice recovered well for the first 4 weeks after RYGB and there were no complications and mortality immediately associated with the surgery. However, two WT mice with RYGB which already passed the nadir of maximal weight loss and started regaining some weight died unexpectedly at 39 and 45 days after surgery for unknown reasons (autopsy inconclusive) and were not included in the analyses. Mice with sham surgery of both genotypes continued to gain weight and fat mass. Three of these morbidly obese mice were euthanized briefly before the 20-week termination point after a short and steep decline in body weight and they are included in all analyses, except for terminal measures of liver weight, fat pad weight, plasma glucose, plasma insulin, and HOMA-IR.

RYGB in WT mice resulted in rapid weight loss with a nadir around 3–5 weeks, with only moderate regain until termination of the study, while sham surgery led to only a small and transient loss followed by continuous further body weight gain (Fig. 1b). All except one WT mice exhibited at least 8% weight loss 20 weeks after RYGB surgery. Remarkably, one WT mouse which started at 40.9 g body weight and responded normally to surgery by reducing body weight to 30.1 g (− 26%) at 6 weeks regained all lost weight and more from week 6–20 to end up with an 11% weight gain relative to preoperative body weight (see outlier in Fig. 1c, d and Fig. 2d, e). Autopsy revealed no anomalies of RYGB surgery. Compared to sham surgery, WT mice with RYGB weighed 35% less (*p* < 0.001) at the end of the study (Fig. 1a, d).

Contrary to the hypothesis, TGR5-KO mice followed more or less the same pattern, so that there were no significant differences in body weight relative to preoperative levels at any time point (Fig. 1a, b). However, body weight and fat mass gain over the 20 weeks after surgery was less in KO mice with sham surgery. As a result, RYGB-induced weight loss relative to sham surgery was slightly but not significantly reduced (Fig. 1c, d), and RYGB-induced loss of fat mass, but not a change in adiposity index, was significantly reduced in TGR5^−/−^ mice (Fig. 2d, e).

Calorie restriction-induced weight matching was less effective in reducing the adiposity index compared to RYGB in both genotypes (Fig. 2c). Analysis of individual fat pad weights at 20 weeks showed slightly but not significantly smaller inguinal and retroperitoneal fat pads after RYGB in WT compared with KO mice (Supplementary Fig. S2).

**Fig. 3:**
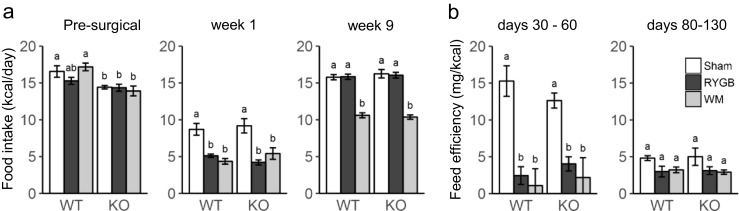
Effect of RYGB, sham surgery, or caloric restriction (to match weight after RYGB) on food intake in TGR5^−/−^ (KO) and wildtype (WT) mice. All mice were on a two-choice diet consisting of high-fat and regular (low-fat) chow. **a** Average daily total food intake before surgery and in week 1 and week 9 after surgery for mice with sham surgery, RYGB, and weight matching. **b** Feeding efficiency for the period with rapid weight gain (days 30–80) and the subsequent period with moderate weight gain of sham-operated mice. Means ± SEM, *n* = 6–11 mice. Bars that do not share the same letters are significantly different from each other (*p* < 0.05, pairwise *t* tests with Benjamini-Hochberg correction, FDR = 0.05)

Food intake was generally slightly lower in TGR5^−/−^ compared to WT mice before surgery (F[1, 48] = 18.7, *p* < 0.001) (Fig. 3a). During the first week after surgery, food intake was similarly suppressed in the two genotypes, but significantly more so after RYGB compared with sham surgery. Food intake after RYGB recovered within 2 weeks (not shown) and at 9 weeks was not significantly different from baseline for either genotype. In weight-matched mice, the amount of food necessary to maintain body weight of the respective RYGB groups was significantly less than food intake of RYGB mice and similar for the two genotypes. Feed efficiency (defined as weight gain per kcal ingested) was significantly lower in RYGB and weight-matched compared to sham-operated mice during the early period when sham mice rapidly gained weight (days 30–60), but not during the latter part of the study when their weight gain had plateaued (days 80–130, Fig. 3b). There were no significant genotype effects on feed efficiency at any time period. In line with our previous observations [32], preference for chow slightly but significantly increased at 1 week but not 9 weeks after RYGB compared to sham surgery in both genotypes (Supplementary Fig. S3). Before and during the first week after surgery, TGR5^−/−^ mice preferred chow slightly less than WT mice.

**Fig. 4:**
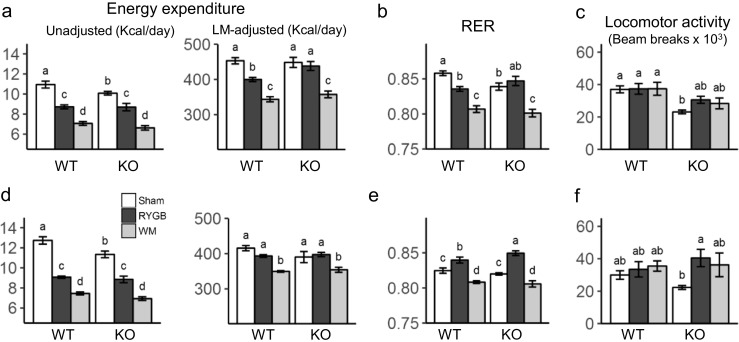
Effect of RYGB on energy expenditure. Effect of RYGB, sham surgery, or caloric restriction (to match weight after RYGB) on energy expenditure (**a**, **d**), respiratory exchange ratio (**b**, **e**), and locomotor activity (**c**, **f**) in TGR5^−/−^ (KO) and wildtype (WT) mice as measured 3 weeks (**a**–**c**) and 17 weeks (**d**–**f**) after surgery in metabolic chambers at thermoneutrality (29 °C). Means ± SEM, *n* = 6–11 mice per group. Bars that do not share the same letters are significantly different from each other (*p* < 0.05, pairwise *t* tests with Benjamini-Hochberg correction, FDR = 0.05)

Total daily energy expenditure at thermoneutrality (29 °C) unadjusted for body weight or composition was highest in sham-operated and lowest in weight-matched mice, with RYGB in the middle, and this was similar for both genotypes and at both 3 weeks (Fig. 4a) and 17 weeks (Fig. 4d). Given the similar body mass of RYGB and weight-matched, but much higher body mass of sham-operated mice, adjusting energy expenditure for lean body mass (Fig. 4a, d) or total body mass (Supplementary Fig. S4) reduced or reversed most of the differences between sham and RYGB mice, but left intact the significantly higher energy expenditure of RYGB vs. weight-matched mice. Importantly, at 3 weeks after surgery, just before RYGB mice reached the nadir of weight loss, they had significantly reduced energy expenditure compared with sham-WT, but not sham-KO mice (Fig. 4a). This genotype-specific effect was no longer observed at 17 weeks after surgery (Fig. 4d). At both time points and for both genotypes, RYGB mice had higher energy expenditure than weight-matched mice. Energy expenditure measured at room temperature (23 °C) essentially followed similar trends (Supplementary Fig. S5). However, energy expenditure corrected for lean body mass in RYGB mice was significantly higher in KO vs. WT mice at both time points and it was lower in RYGB vs. weight-matched mice at 17 weeks.

Respiratory exchange rate (RER) measured at thermoneutrality was significantly higher in RYGB compared to weight-matched mice for both genotypes at both 3 and 17 weeks after surgery (Fig. 4b, e). There was a small but significant genotype effect, in that RER was higher at 17 weeks after RYGB in KO vs. WT mice.

Finally, locomotor activity as assessed in the metabolic chambers at thermoneutrality did not show significant differences between the surgical groups in the WT mice. However, sham-operated KO mice were significantly less active than their WT counterparts at 3 weeks after surgery and KO mice with RYGB at 17 weeks after surgery (Fig. 4c, f).

### Similar RYGB-Induced Improvements of Glycemic Control in both Genotypes in the Face of Slightly Lower Insulin Sensitivity in Sham-Operated Obese TGR5^−/−^ Mice

**Fig. 5:**
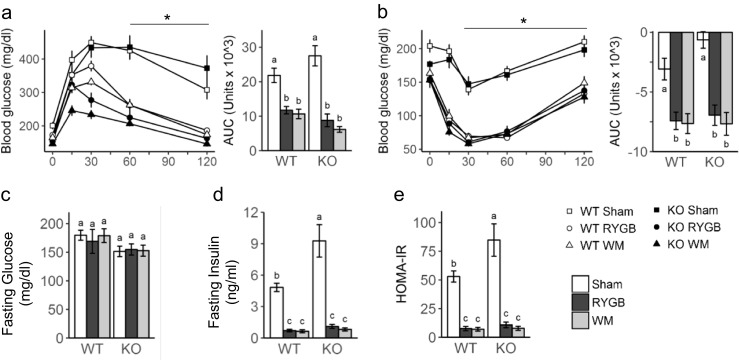
Effect of RYGB on glucose homeostasis. Effect of RYGB, sham surgery, or caloric restriction (to match weight after RYGB) on glucose tolerance, 4 weeks after surgery (**a**), insulin tolerance, 6 weeks after surgery (**b**), fasting glucose (**c**), fasting insulin (**d**), and HOMA insulin resistance (**e**), 20 weeks after surgery in TGR5^−/−^ (KO) and wildtype (WT) mice . Means ± SEM, *n* = 6–11 mice per group. *p < 0.05, RYGB vs. sham for both genotypes. Bars that do not share the same letters are significantly different from each other (*p* < 0.05, pairwise *t* tests with Benjamini-Hochberg correction, FDR = 0.05)

Glucose tolerance at 4 weeks and exogenous insulin effectiveness at 6 weeks after surgery were similarly impaired in sham-operated mice and improved after RYGB in both genotypes (Fig. 5a, b). Fasting blood glucose was slightly but significantly lower in TGR5^−/−^ compared with WT mice at all time points (main effect of genotype at 4 weeks F[1, 48] = 19.84, *p* < 0.001; 6 weeks F [1, 48] = 4.81, *p* < 0.05; 20 weeks F [35, 37] = 5.25, *p* < 0.05) (Fig. 5c). Fasting insulin measured at termination of the study was significantly higher in sham-operated KO compared with WT mice, but was reduced to the same low levels by RYGB and weight matching in both genotypes (Fig. 5d). Similarly, HOMA insulin resistance was significantly higher in sham-operated KO compared with WT mice but was similarly reduced by RYGB and weight matching in both genotypes (Fig. 5e). Importantly, weight matching completely mimicked all beneficial effects on glycemic control in both genotypes.

### RYGB Similarly Improves Liver Weight in WT and KO Mice

**Fig. 6:**
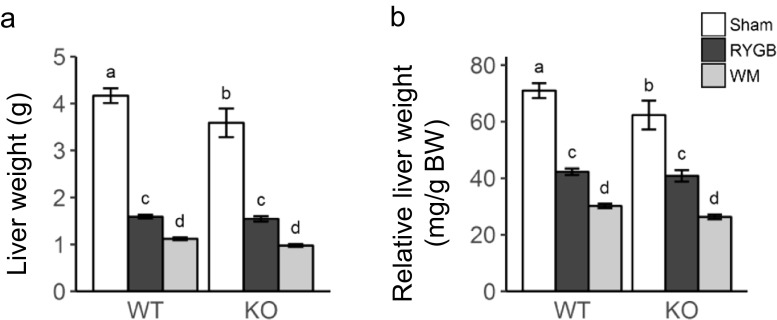
Effect of RYGB, sham surgery, or caloric restriction (to match weight after RYGB) on absolute and relative liver weight. Liver weight was measured 20 weeks after surgery. Means ± SEM, *n* = 6–9 mice per group. Bars that do not share the same letters are significantly different from each other (*p* < 0.05, pairwise *t* tests with Benjamini-Hochberg correction, FDR = 0.05)

Twenty weeks after RYGB surgery, liver weight and liver to total body weight ratio, which likely reflects liver fat content, was significantly lower in RYGB vs. sham-operated obese mice in both genotypes (Fig. 6a, b), but note that three of the sham-operated WT mice died before termination and are not included. Reduction in liver weight and liver to total body weight ratio was significantly higher in mice that lost the same amount of body weight through calorie restriction for both genotypes.

## Discussion

There is a rapidly growing literature demonstrating effects of bile acids on metabolic regulation through both the TGR5 and FXR receptors [33]. Specifically, bile acid signaling through TGR5 can stimulate thermogenic activity of BAT [17], browning of WAT [14], GLP-1 secretion from intestinal L-cells [12], and insulin secretion [13]. Bile acid signaling through the FXR receptor in liver, muscle, and pancreas has also been demonstrated to modulate insulin secretion and sensitivity [15]. Since bariatric surgeries increase total circulating bile acids and change bile acid patterns in humans [20–26] and rodents [9, 11, 27], changes in bile acid signaling are therefore a plausible mechanism for the greatly beneficial effects of bariatric surgeries on body weight and glycemic control.

However, contrary to expectations, we show here that bile acid signaling through TGR5 is not a critical mechanism for the beneficial effects of RYGB in a mouse model of high-fat diet-induced obesity. TGR5-KO mice showed similar loss of body weight and body fat and similar improvements in glycemic control and liver weight compared with sham-operated mice. Our RYGB surgery model produced sustained body weight loss for up to 20 weeks, despite continued ingestion of mainly high-fat diet. Although there was some weight regain, average body weight stayed significantly below pre-surgical levels for the entire 20 weeks for both genotypes.

We have not measured plasma bile acid concentrations in the present study and it is possible that RYGB in the mouse does not result in similar increases of taurine-conjugated bile acids as reported for VSG [9]. At 14 weeks after VSG, muricholic acids (α, β, and ω), particularly their taurine-conjugated forms as well as cholic acid and deoxycholic acid, showed the greatest increases compared to sham surgery [9]. In a mouse model of RYGB, these bile acid species were only modestly and not significantly increased 8 weeks after RYGB under fasting conditions [28]. Therefore, it is possible that the failure of TGR5 deficiency to prevent the beneficial effects of RYGB in the mouse is due to a lack of increased bile acid concentrations. However, this needs further investigation, as in most human studies, total serum bile acids are increased after RYGB.

In terms of beneficial effects on body weight, a previous study found that bile acid signaling through the TGR5 receptor is required for the beneficial effects of VSG [9]. In that study, which used the same mouse strain (C57BL/6J) and high-fat diet (60%) as our study, body weight of TGR5-KO mice subjected to VSG was not significantly different from WT controls up to about 9 weeks after surgery, but then rapidly increased to join the body weight level of their sham-operated counterparts at 12–14 weeks after surgery. In other words, TGR5-signaling was not required for the beneficial effects of VSG up to 9 weeks, but was required for longer-term weight maintenance. However, another study using the same background mouse strain (C57BL/6J), but a slightly different high-fat diet (45%) and a post-surgical observation period of 24 weeks [11], was unable to replicate this “jump” in body weight of TGR5-KO mice at 9 weeks after surgery observed by Ding et al. Our results with RYGB were also unable to replicate the Ding et al. study but agree with the McGavigan et al. study in that the body weight curves were identical between genotypes. While the shorter duration of high-fat exposure and the lower fat content of the diet resulting in lower body weights at the time of surgery may have contributed to the different outcome between the two VSG studies, this cannot be the reason for the different outcome of our RYGB study. However, it is important to note that one major difference between our RYGB and the two VSG studies is the rate of weight regain starting 2–3 weeks after surgery. While weight regain of WT mice is substantial in both VSG studies and body weight reaches preoperative levels after only 10–14 weeks, weight regain after RYGB is minimal and on average, body weight stays clearly below preoperative levels. In a direct comparison of the two surgeries in our own laboratory, we have recently shown this much greater long-term efficacy of RYGB to lower body weight [30].

As we have shown previously, RYGB seems to achieve its body weight lowering effects in WT mice by decreasing food intake at least initially and by increasing energy expenditure relative to the weight-matched controls. Here, we show this to be similar for both genotypes. Ding et al. [9] reported that WT-VSG mice were twice as physically active and expended about 30% more energy than WT-sham mice and that these increases were abolished in the KO-VSG mice. When adjusting energy expenditure for total body mass, we also found WT-RYGB mice to expend significantly more energy than WT-sham mice at 17 weeks after surgery, but this difference was similar in WT and TGR5^−^/^−^ mice. When adjusting for lean body mass, which is highly recommended for animals with different body weight and body composition, we did generally not find differences in energy expenditure between WT-RYGB and WT-sham mice in either genotype. Also, unlike VSG in the Ding et al. study, RYGB did not increase locomotor activity in WT mice and there were no major genotype effects. The only significant genotype effect was a slightly lower RER in TGR5^−/−^ sham-operated mice at 3 weeks and a slightly higher RER of TGR5^−/−^ mice with RYGB at 17 weeks. However, the size of these effects is small compared to the larger and highly significant reduction of RER in weight-matched vs. RYGB mice in both genotypes. The latter suggests that a weight loss-independent mechanism of preferential carbohydrate oxidation is specifically at work after RYGB.

RYGB greatly improved glucose tolerance, fasting insulin, and insulin sensitivity in both genotypes compared to sham surgery. This is in contrast to previous studies with VSG, which found these markers for glycemic control to be improved only in WT but not KO mice [9]. Using euglycemic hyperinsulinemic clamp methodology, Ding et al. also reported that the significantly higher glucose infusion rate and lower hepatic glucose production seen in WT mice after VSG were absent in TGR5^−/−^ mice [9]. At face value, the findings suggest that while important after VSG, TGR5-signaling is not required for improvements in glycemic control after RYGB. The only genotype differences in our study were lower fasting glucose and higher fasting insulin in KO mice when compared to WT.

Weight matching to RYGB was just as effective as RYGB in improving glucose tolerance, fasting insulin levels, and insulin sensitivity/resistance. This is in agreement with a number of clinical studies showing that pair-feeding non-surgical obese patients with very low calorie diet for up to 20 days resulted in similar improvements in glycemic control [34–36]. Although this does not rule out participation of weight loss-independent mechanisms for improvement of glycemic control after RYGB, it suggests that the major effect of bariatric surgeries is due to weight loss [37].

There are some limitations of the present study. We did not measure serum bile acid concentrations and we did not use gold standard techniques such as whole body clamps to characterize changes in glycemic control. Furthermore, because TGR5 was deleted globally, it is possible that tissue-specific effects canceled each other. Also, as is a common problem to germline knockouts, there may have been compensatory changes during development which could make interpretation difficult. Therefore, the study should be repeated in tissue-specific, inducible knockout, or knockdown models.

## Electronic supplementary material

ESM 1 (PDF 276 kb)
