# Supplementary material for: Roux-en-Y Gastric Bypass Surgery-Induced Weight Loss and Metabolic Improvements Are Similar in TGR5-Deficient and Wildtype Mice
Source: Obes Surg. 2018 May 16;28(10):3227–36. doi: 10.1007/s11695-018-3297-6 (PMC6153575; doi:10.1007/s11695-018-3297-6)
Supplement: Supplementary file 1 — (PDF 276 kb) [file 11695_2018_3297_MOESM1_ESM.pdf]

Supplementary Figures

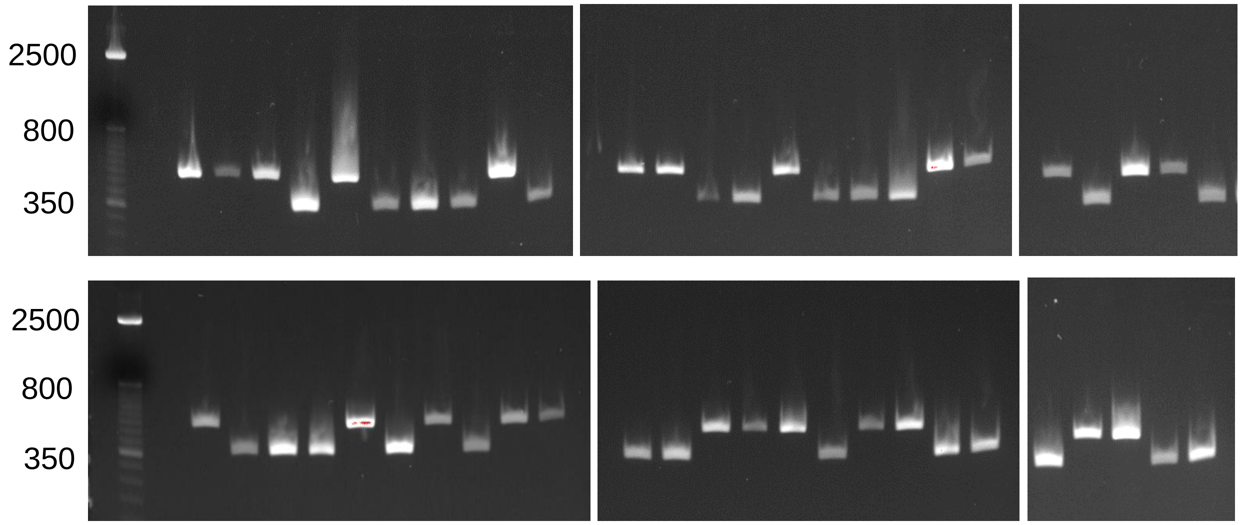

Supplementary Fig. S1. Genotyping of the 25 WT and 25 TGR5$^{-/-}$ mice. Tail biopsies, taken at time of tissue harvest, were incubated at 95 °C in lysis solution for 1hr. Neutralization buffer was added, and tails were stored at -20 °C until time of genotyping. The PCR sample consisted of a 25µl volume containing 1µl of each of three primers (Gpbar1 Fwd: ctacgctagcgacagcacatt atcactgaggctttg ; Gpbar1 Rev: tggccagttactgtcctctcttg ; Neo Primer: cgccccgactgcatctgcgtgtt). Each reaction also contained AmpliTaq Gold 360 Master Mix and 360 GC Enhancer (Applied Biosystems by Thermo Fisher Scientific). The following PCR conditions were applied: 10 min, 95 °C initial denaturation; 30 s, 95 °C cyclic denaturation; 30 s, 58 °C cyclic annealing; 30s, 72 °C cyclic elongation for a total of 35 cycles, followed by a 7 min 72 °C elongation step. PCR amplification products were analyzed by agarose gel electrophoresis. KO band are detected at 503 base pairs and WT band is detected at 363 base pairs.

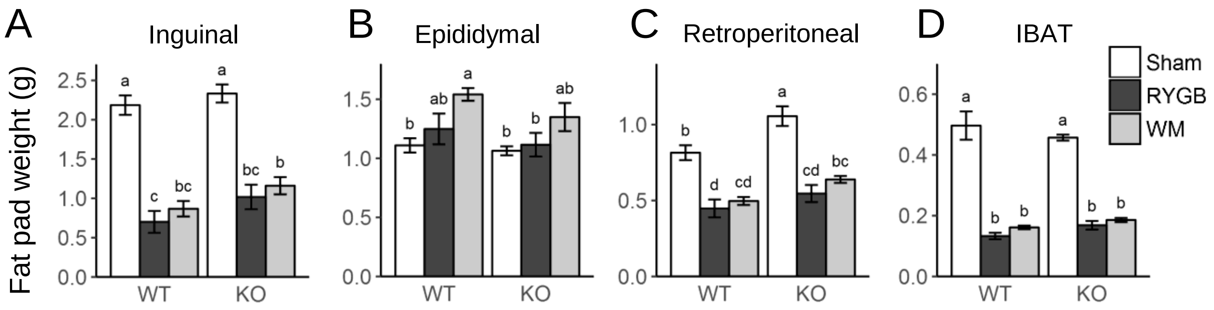

Supplementary Fig. S2. Effect of RYGB on fat pad weights. Effect of RYGB, sham surgery, or caloric restriction (to match weight after RYGB) on weight of inguinal (A), epididymal (B), and retroperitoneal (C) white adipose tissue pads and on interscapular brown fat pad (D). Means ± SEM, n = 6-11 mice. Bars that do not share the same letters are significantly different from each other ($p < 0.05$, pairwise t-tests with Benjamini-Hochberg correction, FDR = 0.05).

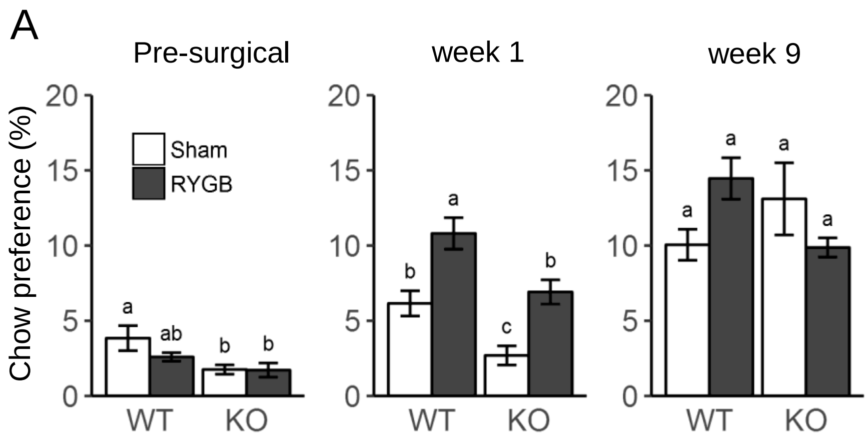

Supplementary Fig. S3. Effect of RYGB, sham surgery, or caloric restriction (to match weight after RYGB) on food choice in TGR5$^{-/-}$ (KO) and wildtype (WT) mice. All mice were on a two choice diet consisting of high-fat and regular (low-fat) chow. Percent chow preference was calculated by the formula Kcal chow/Kcal total x 100. Bars that do not share the same letters are significantly different from each other ($p < 0.05$, pairwise t-tests with Benjamini-Hochberg correction, FDR = 0.05).

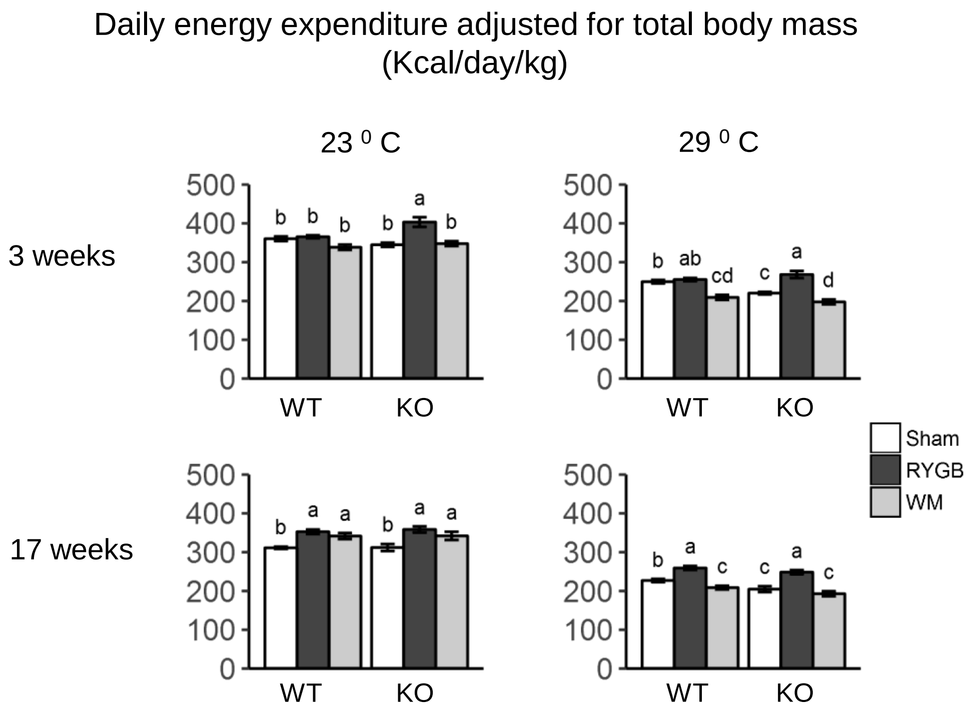

Supplementary Fig. S4. Effect of RYGB, sham surgery, or caloric restriction (to match weight after RYGB) on daily energy expenditure adjusted for total body mass. Energy expenditure was measured at 3 and 17 weeks after surgery and at both room temperature (23 ^{0} C, average of 3 days) and thermoneutrality (29 ^{0} C, average of 3 days). Means ± SEM, n = 6-11 mice per group. Bars that do not share the same letters are significantly different from each other ($p < 0.05$, pairwise t-tests with Benjamini-Hochberg correction, FDR = 0.05).

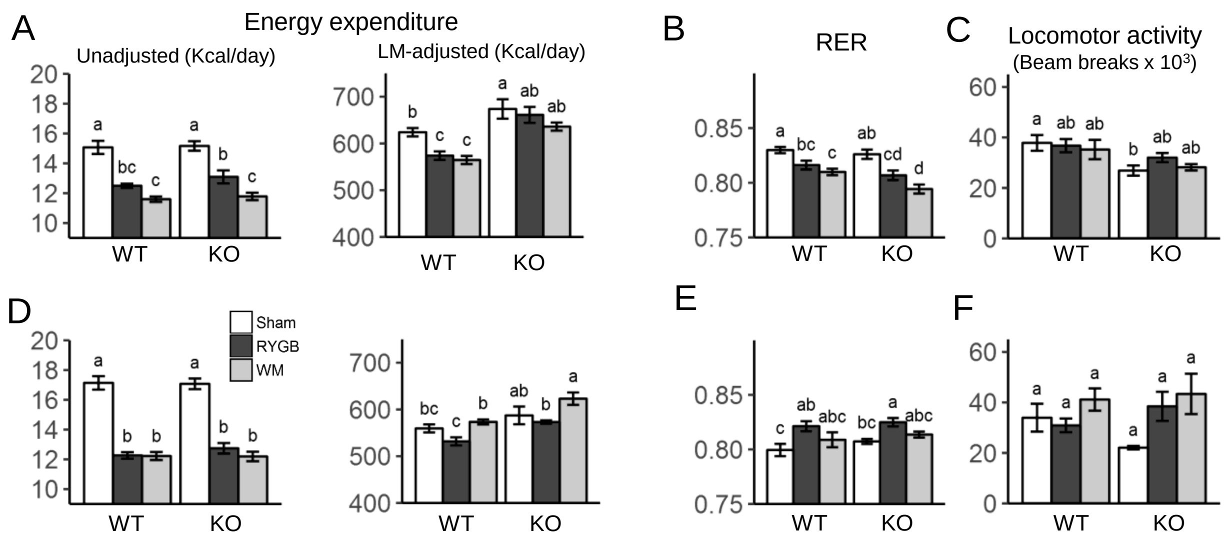

Supplementary Fig. S5. Effect of RYGB on energy expenditure measured at room temperature (23 ^{0} C). Effect of RYGB, sham surgery, or caloric restriction (to match weight after RYGB) on energy expenditure (**A, D**), respiratory exchange ratio (**B, E**), and locomotor activity (**C, F**) in TGR5$^{-/-}$ (KO) and wildtype (WT) mice as measured 3 weeks (**A-C**) and 17 weeks (**D-F**) after surgery in metabolic chambers at room temperature (23°C). Means ± SEM, n = 6-11 mice per group. Bars that do not share the same letters are significantly different from each other ($p < 0.05$, pairwise t-tests with Benjamini-Hochberg correction, FDR = 0.05).
